# Supplementary material for: Determining respiratory rate from photoplethysmogram and electrocardiogram signals using respiratory quality indices and neural networks
Source: PLoS One. 2021 Apr 8;16(4):e0249843. doi: 10.1371/journal.pone.0249843 (PMC8031461; doi:10.1371/journal.pone.0249843)
Supplement: S1 Table — (PDF) [file pone.0249843.s001.pdf]

S1 Table. Respiratory rate distribution in 20-second segment dataset.

RR Parameter	Mean (Min-Max) in BrPM	Median (Q1-Q3) in BrPM
True RR	17.26 (8.01-34.96)	16.11 (13.98-20.03)
ECG-BW RR	20.87 (8.89-36.11)	20.78 (18.34-23.31)
PPG-BW RR	18.91 (5.08-33.50)	18.48 (16.03-21.26)
ECG-AM RR	22.46 (6.23-65.43)	21.69 (18.32-25.93)
PPG-AM RR	24.51 (5.60-70.00)	23.35 (18.86-29.32)
ECG-FM RR	17.30 (2.22-30.00)	17.15 (15.00-20.00)
PPG-FM RR	18.98 (3.33-30.00)	18.95 (16.36-21.43)
