# Supplementary material for: Determining respiratory rate from photoplethysmogram and electrocardiogram signals using respiratory quality indices and neural networks
Source: PLoS One. 2021 Apr 8;16(4):e0249843. doi: 10.1371/journal.pone.0249843 (PMC8031461; doi:10.1371/journal.pone.0249843)
Supplement: S2 Table — (PDF) [file pone.0249843.s002.pdf]

S2 Table. Respiratory rate distribution in 30-second segment dataset.

RR Parameter	Mean (Min-Max) in BrPM	Median (Q1-Q3) in BrPM
True RR	17.07 (8.00-34.90)	16.03 (13.00-19.84)
ECG-BW RR	20.66 (10.71-32.39)	20.64 (18.43-22.89)
PPG-BW RR	18.68 (9.47-30.36)	18.27 (15.97-20.94)
ECG-AM RR	21.94 (5.46-61.40)	21.24 (18.13-25.06)
PPG-AM RR	23.76 (7.58-64.60)	22.37 (18.55-28.18)
ECG-FM RR	16.84 (3.33-30.00)	16.80 (14.59-19.09)
PPG-FM RR	17.07 (8.00-34.90)	16.03 (13.99-19.84)
