# Supplementary material for: Determining respiratory rate from photoplethysmogram and electrocardiogram signals using respiratory quality indices and neural networks
Source: PLoS One. 2021 Apr 8;16(4):e0249843. doi: 10.1371/journal.pone.0249843 (PMC8031461; doi:10.1371/journal.pone.0249843)
Supplement: S3 Table — (PDF) [file pone.0249843.s003.pdf]

S3 Table. Respiratory rate distribution in 60-second segment dataset.

RR Parameter	Mean (Min-Max) in BrPM	Median (Q1-Q3) in BrPM
True RR	16.44 (8.05-34.81)	15.77 (13.98-18.59)
ECG-BW RR	20.46 (12.27-30.97)	20.51 (18.59-22.32)
PPG-BW RR	18.16 (11.98-29.67)	17.66 (15.85-20.29)
ECG-AM RR	21.29 (12.15-41.08)	20.66 (17.50-23.99)
PPG-AM RR	22.79 (10.74-61.90)	21.90 (18.33-26.56)
ECG-FM RR	16.60 (4.80-30.00)	16.47 (14.81-18.33)
PPG-FM RR	18.42 (9.47-30.00)	18.46 (16.72-20.00)
