# Supplementary material for: Determining respiratory rate from photoplethysmogram and electrocardiogram signals using respiratory quality indices and neural networks
Source: PLoS One. 2021 Apr 8;16(4):e0249843. doi: 10.1371/journal.pone.0249843 (PMC8031461; doi:10.1371/journal.pone.0249843)
Supplement: Attachment — Submitted filename: Response to Reviewers.pdf [file pone.0249843.s004.pdf]

Response to Comments

Dear Editors and Reviewers,

We would like to thank you for your insightful and professional review of our work. The valuable reflections you presented have assisted us greatly in improving our paper. The manuscript (Number: **PONE-D-21-02517**, Title: **Determining respiratory rate from photoplethysmogram and electrocardiogram signals using respiratory quality indices and neural networks**) has been thoroughly revised according to your comments and recommendations. The primary corrections are as follows:

1. Figures 1-4 in the 'Introduction' section, which illustrated electrocardiogram and photoplethysmogram signals with and without respiratory modulation, have been majorly revised to combine all four figures into a single figure (now Figure 1) with signals overlaid on grid paper with units included.
2. In the 'Results and discussion' section, Figures 6-8, 9-11, and 12-14 have relabelled as Figures 3a-3c, 4a-4c, and 5a-5c, respectively. They have also been significantly reformatted to improve readability, including the addition of titles and consistent axes limits across plots of the same type.
3. In the 'Introduction' section, a table has been added to define abbreviations and acronyms used throughout the manuscript.
4. Supplementary tables illustrating the distribution of true and modulation-derived respiratory rates have been added to the new "Supplementary information" section of the manuscript.
5. Model code and code used for generating figures has been uploaded to the freelly available repository `https://github.com/stephb23/RespiratoryRate/` in accordance with reviewer feedback.
6. Improvements to definitions of functions and acronyms have been made throughout the manuscript.
7. Minor formatting improvements to headings and title page have been made to meet PLOS ONE formatting requirements.

All modifications to the manuscript have been highlighted in yellow. The remainder of this response letter contains a detailed response to every comment received from the editor and reviewer. Please note that all references quoted in this response document are numbered consistently with the main manuscript.

We would like to again extend our sincere gratitude to the editor and reviewers for your valuable feedback on our original manuscript. We look forward to receiving your feedback on our carefully constructed revision.

Yours sincerely,

The Authors.

Editorial Comments

Comment 1: Please ensure that your manuscript meets PLOS ONE's style requirements, including those for file naming. The PLOS ONE style templates can be found at

`https://journals.plos.org/plosone/s/file?id=wjVg/PLOSOne_formatting_sample_main_body.pdf`

`https://journals.plos.org/plosone/s/file?id=ba62/PLOSOne_formatting_sample_title_authors_affiliations.pdf`

Response: Thank you for providing these references. We have improved the formatting of our manuscript in the following ways:

- Initials have been added after the corresponding author's initials.
- Headings and subheadings have been converted to sentence case.
- Figure, table, and algorithm titles have been corrected to bold font.
- Funding information has been removed from the Acknowledgements section.

The manuscript was created using the PLOS ONE LaTeX template provided at `https://journals.plos.org/plosone/s/latex#loc-plos-template`. Upon thoroughly reviewing the references provided by the editor, we believe that we have now met the formatting requirements of PLOS ONE.

Comment 2: Thank you for providing the date(s) when patient medical information was initially recorded. Please also include the date(s) on which your research team accessed the databases/records to obtain the retrospective data used in your study.

Response: Thank you for this feedback. We have added a statement to the "Obtaining data" subsection of the "Methodology" to clarify that data was first accessed in 2019. The introduction of this subsection now reads as follows:

> Data for this work was obtained from the open-source Medical Information Mart for Intensive Care (MIMIC-III) database [22], which features an extremely large number of records from patients admitted to intensive care units (ICUs) between 2001-2016. Data used to conduct this research was first accessed in 2019.

Comment 3: To meet our data availability requirements, please provide the data used for this study. This may be a supplementary table that includes basic demographic information and measurements of the six modulation-extracted respiratory signals.

Please provide a summary table of patient demographics.

Response: Thank you for your guidance regarding meeting data availability requirements. The MIMIC-III waveform database contains many records that are not linked to patient information; therefore demographics information is not available. However, the paper that presented MIMIC-III [22] contains an overview of patient demographics across the entire MIMIC-III database. We have added a reference to this in the "Obtaining data' subsection of our manuscript, as follows:

> Patient demographics are also not attached to many of the waveform records used, however an overview of patient demographics across the entire MIMIC-III database is presented in the original paper describing the database [22].

In response to your feedback, we have created three supplementary tables that show the distribution of true RR compared to the six modulation-derived RRs for the 20-second segment, 30-second segment, and 60-second segment databases. Statistics included are mean, minimum, maximum, median, Q1, and Q3. The three supplementary tables are as follows:

S1 Table. Respiratory rate distribution in 20-second segment dataset.

RR Parameter	Mean (Min-Max) in BrPM	Median (Q1-Q3) in BrPM
True RR	17.26 (8.01-34.96)	16.11 (13.98-20.03)
ECG-BW RR	20.87 (8.89-36.11)	20.78 (18.34-23.31)
PPG-BW RR	18.91 (5.08-33.50)	18.48 (16.03-21.26)
ECG-AM RR	22.46 (6.23-65.43)	21.69 (18.32-25.93)
PPG-AM RR	24.51 (5.60-70.00)	23.35 (18.86-29.32)
ECG-FM RR	17.30 (2.22-30.00)	17.15 (15.00-20.00)
PPG-FM RR	18.98 (3.33-30.00)	18.95 (16.36-21.43)

S2 Table. Respiratory rate distribution in 30-second segment dataset.

RR Parameter	Mean (Min-Max) in BrPM	Median (Q1-Q3) in BrPM
True RR	17.07 (8.00-34.90)	16.03 (13.00-19.84)
ECG-BW RR	20.66 (10.71-32.39)	20.64 (18.43-22.89)
PPG-BW RR	18.68 (9.47-30.36)	18.27 (15.97-20.94)
ECG-AM RR	21.94 (5.46-61.40)	21.24 (18.13-25.06)
PPG-AM RR	23.76 (7.58-64.60)	22.37 (18.55-28.18)
ECG-FM RR	16.84 (3.33-30.00)	16.80 (14.59-19.09)
PPG-FM RR	17.07 (8.00-34.90)	16.03 (13.99-19.84)

S3 Table. Respiratory rate distribution in 60-second segment dataset.

RR Parameter	Mean (Min-Max) in BrPM	Median (Q1-Q3) in BrPM
True RR	16.44 (8.05-34.81)	15.77 (13.98-18.59)
ECG-BW RR	20.46 (12.27-30.97)	20.51 (18.59-22.32)
PPG-BW RR	18.16 (11.98-29.67)	17.66 (15.85-20.29)
ECG-AM RR	21.29 (12.15-41.08)	20.66 (17.50-23.99)
PPG-AM RR	22.79 (10.74-61.90)	21.90 (18.33-26.56)
ECG-FM RR	16.60 (4.80-30.00)	16.47 (14.81-18.33)
PPG-FM RR	18.42 (9.47-30.00)	18.46 (16.72-20.00)

A reference to these supplementary tables has been included at the end of the "Extracting respiratory signals" subsection of the "Methodology". The sentence reads as follows:

Overviews of the distribution of modulation-derived respiratory rates, along with the distribution of true respiratory rates, are presented for the 20-second segment dataset in S1 Table, the 30-second segment dataset in S2 Table, and the 60-second segment dataset in S3 Table.

Comment 4: .Thank you for stating the following in the Acknowledgments Section of your manuscript:

"This work was supported by the Australian Government Research Training Program Scholarship."

We note that you have provided funding information that is not currently declared in your Funding Statement. However, funding information should not appear in the Acknowledgments section or other areas of your manuscript. We will only publish funding information present in the Funding Statement section of the online submission form.

Please remove any funding-related text from the manuscript and let us know how you would like to update your Funding Statement. Currently, your Funding Statement reads as follows:

"The authors received no specific funding for this work."

Please include your amended statements within your cover letter; we will change the online submission form on your behalf.

Response: Thank you for this feedback. We have removed the Acknowledgements section in response to this feedback, as no further acknowledgements were made. We would also like to update the funding statement to state the following:

"This work was supported by the Australian Government Research Training Program Scholarship."

We have also included this request in our revised submission as per editorial instructions.

Comments from Reviewer 1

Comment 1: In "Determining respiratory rate from photoplethysmogram and electrocardiogram signals using respiratory quality indices and neural networks" by Baker, Xiang, and Atkinson, the authors propose a method to approximate Respiratory Rate (RR) from other common signals (ECG, PPG) and based on idealized neural network models fit (using 'Adam Optimization').

A key result appears to be in Table 1 where including RQI lowers errors and increases correlation. I appreciate Table 2, comparison to previous works. This paper overall appears sound. My biggest concerns with lack of clarity, basic definitions of functions (see below), and a seeming lack of care or experience in thinking through figures are disappointing. But hopefully these issues can be fixed.

Response: Thank you for your feedback. We appreciate the time that you have taken to review our manuscript and offer valuable recommendations for improvement. We have modified the paper to improve clarity through making code readily available, better defining functions, and altering the figures to improve readability. These revisions are detailed in our responses to your individual comments below. We hope that the improvements made based on your feedback in the following comments will meet your expectations.

Comment 2: Consistent with PLoS policy, the authors should make all of the code for their models freely available at a public repository.

They should also have the scripts to generate the exact figures and not just the minimal working model.

This to me is the most crucial part of having this paper accepted because aspects of the models that are not easily understood with the current descriptions (see below).

Response: Thank you for this feedback. We have now made the code for our model available in a GitHub repository, along with all code for the generation of the error distribution, Bland Altman, and regression plots. The repository is freely available at `https://github.com/stephb23/RespiratoryRate/`.

Comment 3: All of the acronyms are quite hard to follow. I strongly urge the authors to add a table upfront to define the many acronyms/abbreviations.

Response: Thank you for this feedback. In response to your comment, we have added the following table to the end of the "Introduction" section of the revised manuscript.

Table 1: Acronyms and abbreviations

Abbreviation	Definition
AM	Amplitude modulation
BiLSTM	Bidirectional long short-term memory
BrPM	Breaths per minute
BrTBr	Breath-to-breath
BTB	Beat-to-beat
BW	Baseline wander
DCV	Differential coefficient of variation
ECG	Electrocardiogram
FM	Frequency modulation
HR	Heart rate
ICU	Intensive care unit
LOA	Limit of agreement
LSTM	Long short-term memory
MAE	Mean absolute error
MD	Mean difference
MIMIC	Medical Information Mart for Intensive Care
ML	Machine learning
NN	Neural network
PCC	Pearson's correlation coefficient
PPG	Photoplethysmograph
RMSE	Root mean square error
RQI	Respiratory quality index
RR	Respiratory rate
RSA	Respiratory sinus arrhythmia
SQI	Signal quality index

Aside from adding the above table, we also removed several abbreviations that were defined but not used thereafter in the manuscript. These included ED (emergency department), MEWS (modified early warning score), WFDB (waveform database), BP (blood pressure), and CV (coefficient of variation). By removing these abbreviations that were only used once in the manuscript, we have improved the readability of the overall work.

Comment 4: Eq (5)—(10): what is the function $\sigma()$? Or is this σ just the std. dev from eq (3)? I understand tanh() is commonly used to represent a sigmoidal nonlinear saturation, a commonly used transfer function in neural networks. Also if the variable c_t are matrices, what are the state variables?

Response: Thank you for this feedback. We apologise for not defining the function $\sigma()$ in our original manuscript. In the context of LSTM, $\sigma()$ represents the sigmoid activation function. We have updated the paragraph explaining the terminology for Eqs. (5)-(10) to conclude as follows:

The '•' symbol in Eqs (9-10) represents element-wise matrix multiplication, and the function $\sigma()$ in Eqs (6-8) is the sigmoid activation function, which is defined as $\sigma(z) = \frac{1}{1+e^{-z}}$.

To address further queries regarding the functionality of LSTM networks, we have also included a reference to the original paper where LSTM was proposed to allow the interested reader to delve deeper into the mathematical theory of LSTM. The introductory paragraph of subsection "Neural Network Structure" now concludes as follows:

The mathematical structure of a single forward or backwards pass is described by the following equations, with interested readers referred to the original paper that introduced LSTM for further details regarding mathematical theory [32].

We hope that these modifications effectively clarify our use of LSTM and the mathematical definitions included in our work.

Comment 5: Also I'm not quite familiar with Adam Optimization... the extent of my experience in deep neural nets is that the large # of params begs to use standard gradient decent. I and other readers would greatly appreciate it if the authors please explain these details and say a few words about the optimization algorithm.

Response: Thank you for this feedback. Adam optimization offers several improvements over standard gradient descent. It uses adaptive learning rates to fine tune the steps taken in each training iteration for each parameter, which enhances its ability to converge on a solution quickly and efficiently. Additionally it uses a momentum term that prevents it from becoming 'stuck' at a local minima, ensuring that it will converge on the true minima. To address your comment, we have added the following sentence to the second paragraph of subsection "Neural network structure", including a reference to the original paper for Adam optimization:

These are learnt during training using the Adam optimization algorithm [28], a common optimization algorithm that uses adaptive learning rates and momentum to converge quickly and efficiently on the true optimal solution.

**Comment 6:**	Figures 1–4 need axes title, labels, units, numbers. I've never seen anything so bare unless it was part of a schematic that is part of a larger figure.<br><br>For readability, the authors should consider making these all into 1 figure with labels. In its current form, it is really hard to see anything they are trying to communicate.

Response: Thank you for this feedback. Figures 1-4 were graphical drawings intended to illustrate how baseline wander, amplitude modulation, and frequency modulation modify the ECG and PPG waveforms. While these do not represent actual signals from our database, we agree that clarity could be improved through the addition of axes and labels. As per your recommendation, we have combined the four illustrative figures into a single figure. This figure shows the ECG and PPG signals overlaid on an orange grid (similar to the grid that would be used for a real signal). Axes are labelled as time and voltage with corresponding units. The scale is arbitrary given that these signals are for illustrative purposes only. The updated figure is shown below, and has replaced Figures 1-4 in the manuscript.

Figure 1: **Sample ECG and PPG signals with and without effects of respiratory modulation.** (A) unaffected by respiratory modulation. (B) affected by baseline wander. (C) affected by amplitude modulation. (D) affected by frequency modulation.

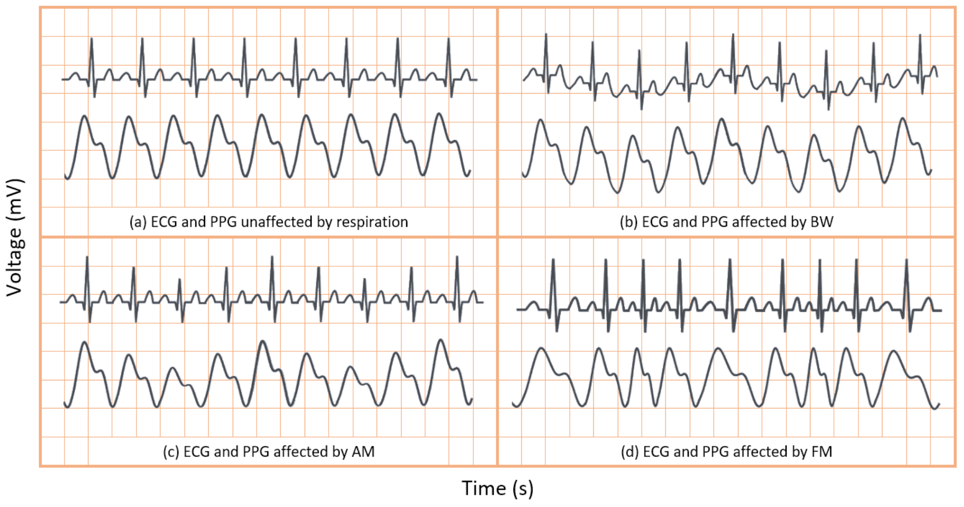

**Comment 7:**	Same comments for Figs 6–8, 9–11, 12–14: please combine the figures; this would make it easier for readers to digest your results.

Response: Thank you for your suggestions for improving the readability of the figures. We have modified the error histograms to be three sub-figures

with fixed horizontal axes limits. As different quantities of data were available for training and testing depending on the segment length, vertical axes were set to appropriate limits to maximise readability of each plot. We have modified our manuscript to indicate that the figures should be presented as Fig. 3a, Fig. 3b, and Fig. 3c, rather than as three separate figures, and have included titles for each sub-figure. The revised figures and their caption are shown below.

Fig 3. Error Histograms for RR Estimation using RR & RQI features derived from: (A) 20-second PPG & ECG segments. (B) 30-second PPG & ECG segments. (C) 60-second PPG & ECG segments.

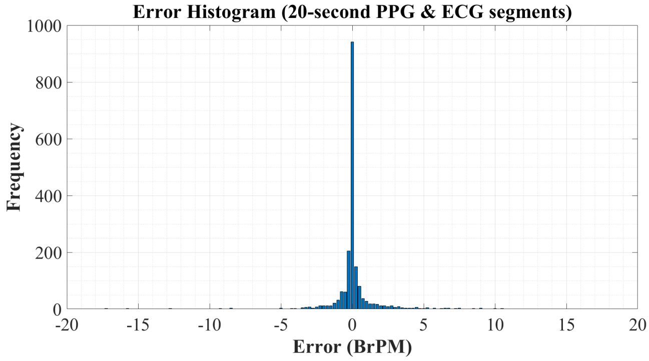

(a)

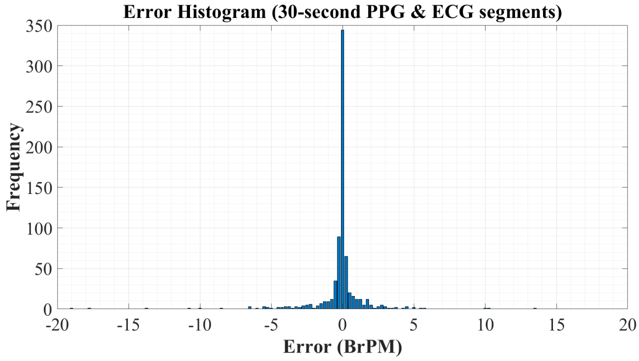

(b)

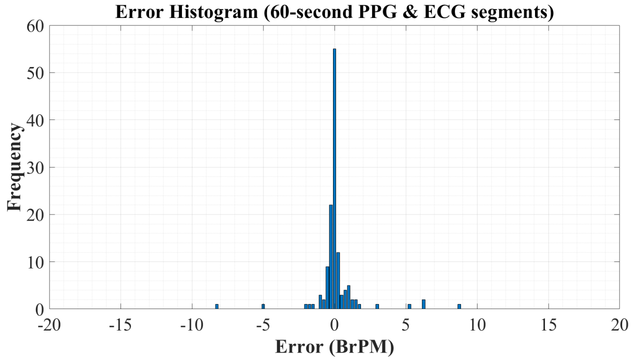

(c)

.

To improve readability of the Bland Altman plots, we have modified each plot to have the same limits for the vertical and horizontal axes,

allowing more direct comparison between the plots. Further, we have added titles to each sub-figure to improve clarity. We have also modified the caption in the manuscript to indicate that these should be presented as three sub-figures - Figs. 4a, 4b, and 4c - rather than three individual figures. The updated figures and their caption are shown overleaf.

Fig 4. Bland Altman Plots for RR Estimation using RR & RQI features derived from: (A) 20-second PPG & ECG segments. (B) 30-second PPG & ECG segments. (C) 60-second PPG & ECG segments.

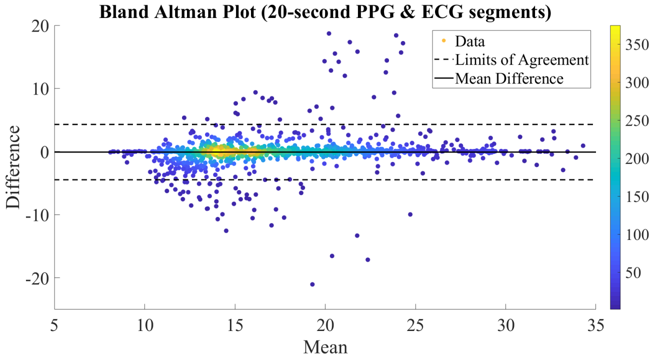

(a)

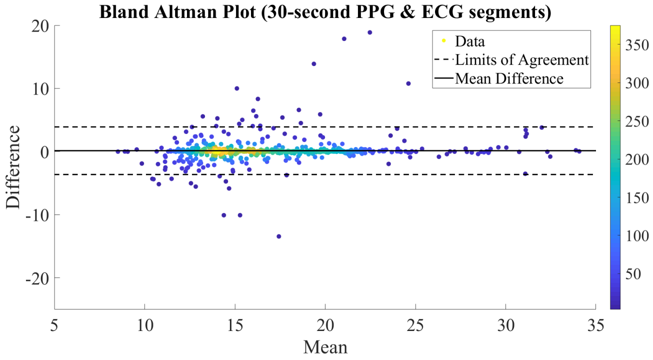

(b)

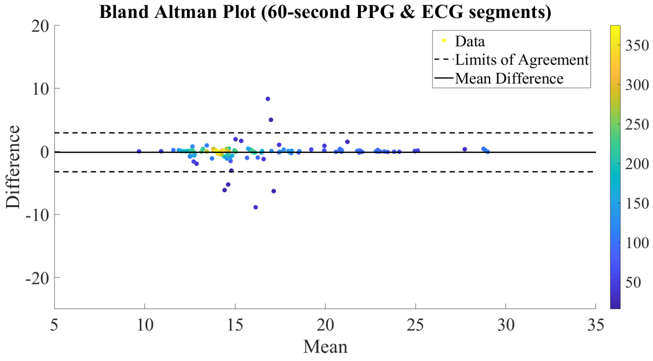

(c)

.

To address the readability of the regression plots, vertical and horizontal axes were again modified to have the same limits across all

three plots. This allows for more direct comparison between the three graphs. Titles have been added for enhanced clarity, and the caption in the manuscript has been updated to reflect the fact that the plots should be presented as three sub-figures, Fig. 5a, Fig. 5b, and Fig. 5c. The updated caption and figures are shown below.

Fig 5. Regression Plots for RR Estimation using RR & RQI features derived from: (A) 20-second PPG & ECG segments. (B) 30-second PPG & ECG segments. (C) 60-second PPG & ECG segments.

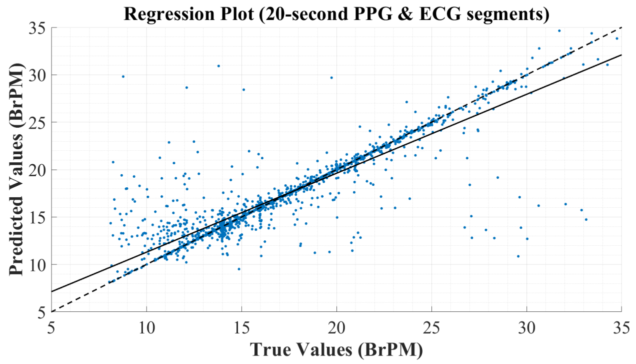

(a)

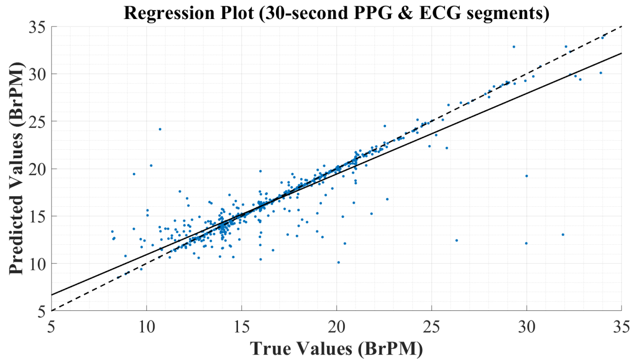

(b)

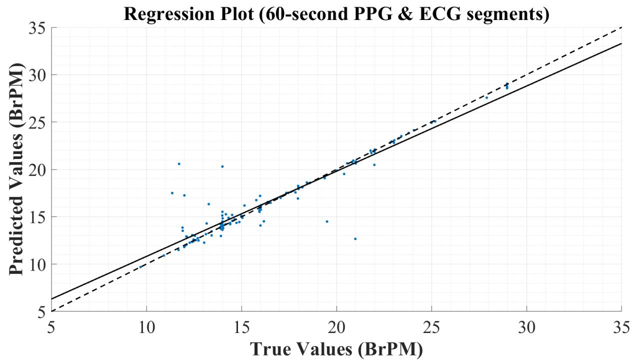

(c)

.

We hope that these modifications to improve the readability of our results meet your expectations.

Comment 8: In Table 2: presumably the other methods [14—19] did not have PCC? If so, that would be important to include; if not, please state this.

Response: Thank you for this question. You are correct in assuming that the previous works included in Table 2 did not provide PCC or comparable correlation statistics. We have modified the introductory paragraph of the subsection "Comparison to previous works" to clarify that PCC would not be available. The full paragraph is as follows, with the final sentence pertaining to the unavailability of PCC.

> The results obtained by our BiLSTM models compare well to previous works when the feature vectors with both modulation-extracted RRs and corresponding RQIs were used, regardless of the segment length that these features were extracted from. This is shown in Table 3. It is clear that the proposed model outperforms the previous state-of-the-art schemes for RR estimation from ECG and PPG signals, achieving significantly better MAE and comparable RMSE. Unfortunately, PCC was not provided by previous works in Table 3 so could not be considered when making comparisons to the literature.

Comment 9: Minor: MAE is not defined in the abstract but all other acronyms are. Mean absolute error defined on line 57. Please define what it is or don't use the abbreviation.

Response: Thank you for your attention to detail. We have now defined MAE as mean absolute error in the abstract, with the full sentence reading as follows:

> We determine that calculating RQIs for modulation-extracted RRs decreased the mean absolute error (MAE) of our NNs by up to 38.17%.

MAE is additionally defined in the acronym table (Table 1) that was added in response to your feedback, and upon first usage in the main body of the article.
